# Correction: Music listening evokes story-like visual imagery with both idiosyncratic and shared content

**DOI:** 10.1371/journal.pone.0317174

**Published:** 2025-01-03

**Authors:** Sarah Hashim, Lauren Stewart, Mats B. Küssner, Diana Omigie

There is an error in Table 1. In the ’Survey 1 (N = 353) of Mexico’ category, the values for the ’Sum’ and ’Proportion’ columns should be revised to ’17’ and ’4.8%’, respectively. Additionally, the ’Sum’ value for ’Other’ should be corrected to ’87’. Please see the corrected Table 1 here.

**Table 1:** Countries of residence of the samples from survey 1 and survey 2.

Country of Residence	Survey 1 (N = 353)		Survey 2 (N = 254)	
	Sum	Proportion	Sum	Proportion
**United Kingdom**	79	22.4%	38	15.0%
**Portugal**	59	16.7%	48	18.9%
**Poland**	48	13.6%	37	14.6%
**Italy**	18	5.1%	16	6.3%
**Canada**	18	5.1%	14	5.5%
**Mexico**	17	4.8%	15	5.9%
**Germany**	14	4.0%	3	1.2%
**Greece**	13	3.7%	12	4.7%
**Other**	87	24.6%	71	28.0%

Table 2 is formatted incorrectly. Please see the correct Table 2 here.

**Table 2:** Thematic framework of prominent higher-order themes (L1) of music-induced visual imagery content (with L2 and L3 codes in descending order of prevalence). Values included beside L1 codes represent their proportions within the entire framework, whereas the two values beside L2 codes reflect their proportions within their higher-order theme (left) and within the entire framework (right).

Level 1	Storytelling (74.3%)				Associations (6.4%)			References (19.3%)	
Level 2	Setting & Location (38.0%, 28.2%)	Characters (27.5%, 20.4%)	Action (17.7%, 13.1%)	Narrative (16.8%, 12.5%)	Abstract & Other (59.9%, 3.8%)	Feelings & Atmospheres (29.6%, 1.9%)	Memories (10.9%, 0.7%)	Other Media (89.5%, 17.2%)	Music (10.5%, 2.0%)
Level 3	Building	Imagery of Self	Communicative Gestures	Plot Lines	Physical Reactions	Emotional Reaction/Feelings	Autobiographical	Medium	Genre
	Urban	Individuals	Idle/Passive	Hardship	Other Senses	Atmosphere/Mood	Episodic	Genres	Composer
	Nature	Royal	Mental	Conflict & Resolution	Cross-Modal			Specific Movie/Game/Show	Characteristics
	Interior & Exterior Features	Heroic	Interaction	Celebratory				Features	Instruments
	Existing Locations	Evildoer	Musical	Inquisitive					
	Imagined Setting	Civilian	Leisure						
	Time References	(Social) Groups	Gait						
	Season	Musical							
	Weather	Animals/Insects							
	Other Objects	Characteristics							
