# Supplementary material for: Music listening evokes story-like visual imagery with both idiosyncratic and shared content
Source: PLoS One. 2023 Oct 26;18(10):e0293412. doi: 10.1371/journal.pone.0293412 (PMC10602345; doi:10.1371/journal.pone.0293412)
Supplement: S1 File — (DOCX) [file pone.0293412.s001.docx]

**Appendices**

**Table A. Independent samples t-tests of prevalence of music-induced visual imagery ratings between those who do and those who do not participate in the visual arts**

|  | **Visual Arts** | | **No Visual Arts** | |  |  |  |
| --- | --- | --- | --- | --- | --- | --- | --- |
|  | **M** | **SD** | **M** | **SD** | ***df*** | ***t*** | ***p*** |
| **All** | 4.63 | 1.23 | 4.00 | 1.40 | 119.5 | 3.79 | **< 0.001** |
| **Happy** | 4.94 | 1.60 | 4.34 | 1.81 | 119.8 | 2.74 | **0.007** |
| **Tender** | 4.87 | 1.56 | 4.03 | 1.85 | 124.68 | 3.88 | **< 0.001** |
| **Fearful** | 5.13 | 1.43 | 4.58 | 1.71 | 125.01 | 2.74 | **0.007** |

Bolded p-values indicate statistical significance at the cut-off 0.05.

**Table B. Independent samples t-tests of vividness of music-induced visual imagery ratings between those who do and those who do not participate in the visual arts**

|  | **Visual Arts** | | **No Visual Arts** | |  |  |  |
| --- | --- | --- | --- | --- | --- | --- | --- |
|  | **M** | **SD** | **M** | **SD** | ***df*** | ***t*** | ***p*** |
| **All** | 4.31 | 1.21 | 3.74 | 1.44 | 124.2 | 3.37 | **< 0.001** |
| **Happy** | 4.61 | 1.57 | 4.15 | 1.81 | 121.38 | 2.12 | **0.036** |
| **Tender** | 4.37 | 1.54 | 3.88 | 1.93 | 131.36 | 2.24 | **0.027** |
| **Fearful** | 4.68 | 1.54 | 4.19 | 1.74 | 120.08 | 2.29 | **0.023** |

Bolded p-values indicate statistical significance at the cut-off 0.05.

**Table C. Banded within-participant Jaccard consistency values across levels 2 and 3 and across music excerpts**

|  | Within-Participants | | | | | | | | |
| --- | --- | --- | --- | --- | --- | --- | --- | --- | --- |
|  | Level 3 (% of overall) | | | | Level 2 (% of overall) | | | | |
|  | **Overall** | Happy | Tender | Fearful | **Overall** | Happy | Tender | Fearful |  |
| 0% | **300 (41.7%)** | 104 (43.3%) | 112 (46.7%) | 84 (35.0%) | **195 (27.1%)** | 59 (24.6%) | 82 (34.2%) | 54 (22.5%) |  |
| 0.01–19.99% | **99 (13.8%)** | 30 (12.5%) | 31 (12.9%) | 38 (15.8%) | **79 (11.0%)** | 22 (9.2%) | 16 (6.7%) | 41 (17.1%) |  |
| 20–39.99% | **178 (24.7%)** | 55 (22.9%) | 54 (22.5%) | 69 (28.7%) | **255 (35.4%)** | 89 (37.1%) | 72 (30.0%) | 94 (39.2%) |  |
| 40–59.99% | **84 (11.7%)** | 26 (10.8%) | 27 (11.2%) | 31 (12.9%) | **122 (16.9%)** | 44 (18.3%) | 42 (17.5%) | 36 (15.0%) |  |
| 60–79.99% | **35 (4.9%)** | 14 (5.8%) | 11 (4.6%) | 10 (4.2%) | **40 (5.6%)** | 15 (6.2%) | 19 (7.9%) | 6 (2.5%) |  |
| 80–99.99% | **1 (0.1%)** | 0 (0.0%) | 1 (0.4%) | 0 (0.0%) | **1 (0.1%)** | 0 (0.0%) | 1 (0.4%) | 0 (0.0%) |  |
| 100% | **23 (3.2%)** | 11 (4.6%) | 4 (1.7%) | 8 (3.3%) | **28 (3.9%)** | 11 (4.6%) | 8 (3.3%) | 9 (3.8%) |  |

Within-participant total % calculated from N = 720. From total % for music excerpts were computed using each individual N sizes; N_Happy_ = 240, N_Tender_ = 240, N_Fearful_ = 240.

**Table D. Banded across-participant Jaccard consistency values across levels 2 and 3 and across music excerpts**

|  | Across-Participants | | | | | | | | |
| --- | --- | --- | --- | --- | --- | --- | --- | --- | --- |
|  | Level 3 (% of overall) | | | | Level 2 (% of overall) | | | | |
|  | **Overall** | Happy | Tender | Fearful | **Overall** | Happy | Tender | Fearful |  |
| 0% | **0 (0.00%)** | 0 (0.00%) | 0 (0.00%) | 0 (0.00%) | **0 (0.00%)** | 0 (0.00%) | 0 (0.00%) | 0 (0.00%) |  |
| 0.01–4.99% | **126 (11.9%)** | 71 (20.1%) | 33 (9.3%) | 22 (6.2%) | **33 (3.1%)** | 19 (5.4%) | 9 (2.5%) | 5 (1.4%) |  |
| 5–9.99% | **432 (40.8%)** | 169 (47.9%) | 148 (41.9%) | 115 (32.6%) | **130 (12.3%)** | 49 (13.9%) | 24 (6.8%) | 57 (16.1%) |  |
| 10–14.99% | **402 (38.0%)** | 101 (28.6%) | 164 (46.5%) | 137 (38.8%) | **242 (22.9%)** | 43 (12.2%) | 98 (27.8%) | 101 (28.6%) |  |
| 15–19.99% | **94 (8.9%)** | 12 (3.4%) | 8 (2.3%) | 74 (21.0%) | **276 (26.1%)** | 87 (24.6%) | 91 (25.8%) | 98 (27.8%) |  |
| 20–24.99% | **5 (0.5%)** | - | - | 5 (1.4%) | **248 (23.4%)** | 80 (22.7%) | 91 (25.8%) | 77 (21.8%) |  |
| 25-29.99% | **-** | - | - | - | **99 (9.3%)** | 60 (17.0%) | 25 (7.1%) | 14 (4.0%) |  |
| 30-34.99% | **-** | - | - | - | **25 (2.4%)** | 9 (2.5%) | 15 (4.2%) | 1 (0.3%) |  |
| 35-40% | **-** | - | - | - | **6 (0.6%)** | 6 (1.7%) | - | - |  |

Across-participant total % were calculated from N = 1059. From total % for music excerpts were computed using each individual N sizes; N_Happy_ = 353, N_Tender_ = 353, N_Fearful_ = 353.
