# Supplementary material for: Music listening evokes story-like visual imagery with both idiosyncratic and shared content
Source: PLoS One. 2023 Oct 26;18(10):e0293412. doi: 10.1371/journal.pone.0293412 (PMC10602345; doi:10.1371/journal.pone.0293412)
Supplement: S2 File — (DOCX) [file pone.0293412.s002.docx]

**Supplementary Information**

1. **Supplementary figures**


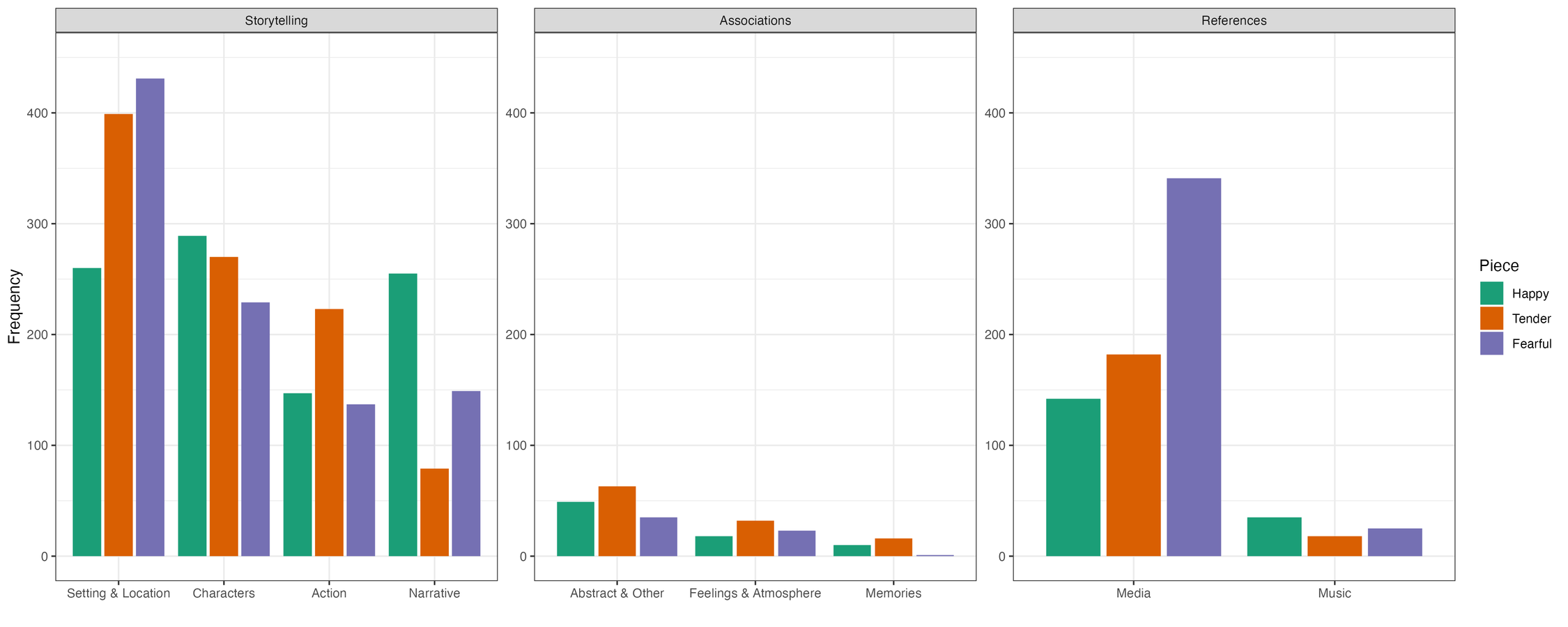


**Fig A. Level 2 content of visual imagery frequencies divided by excerpt**


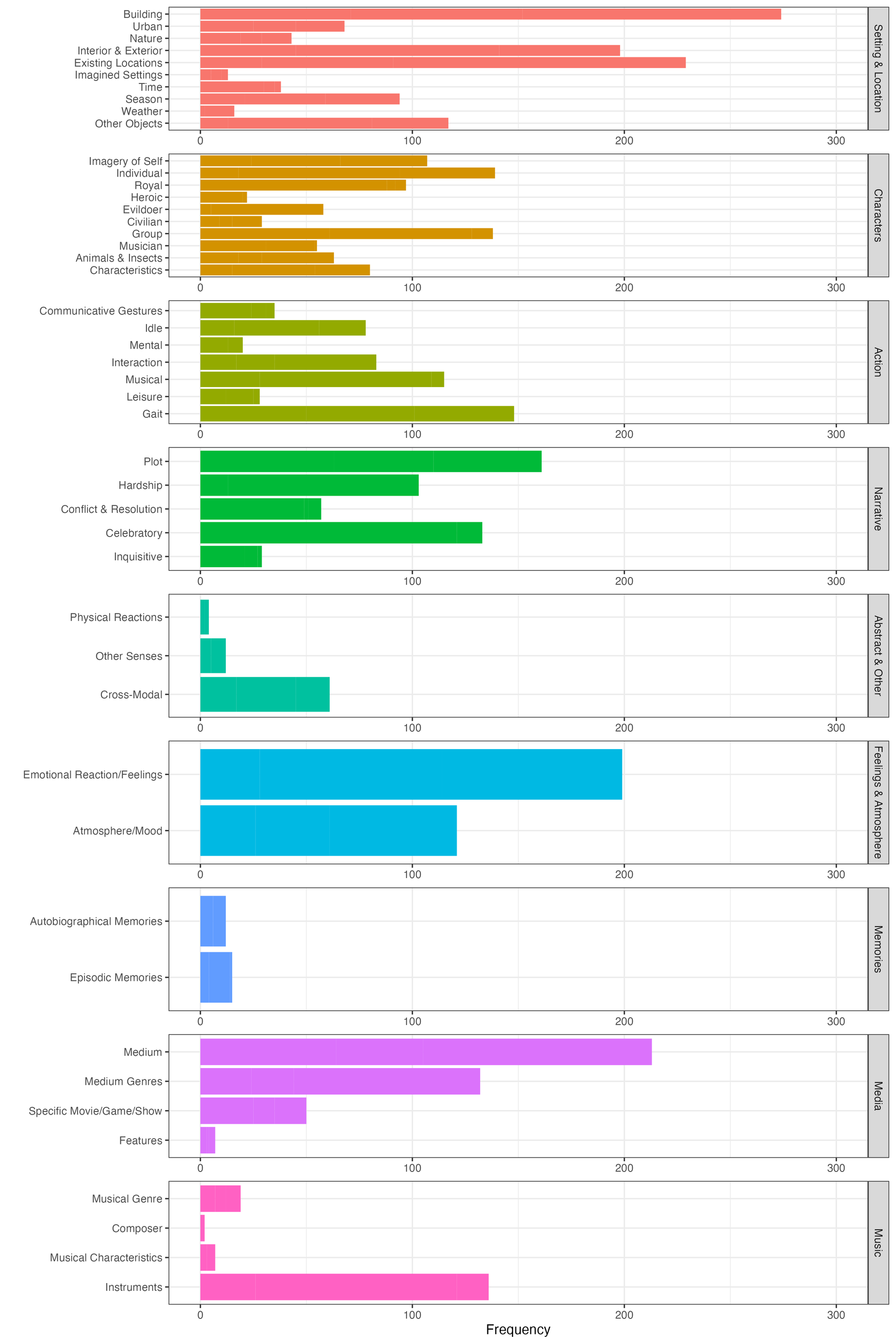


**Fig B. Frequencies of level 3 visual imagery content, segmented by level 2 groupings**


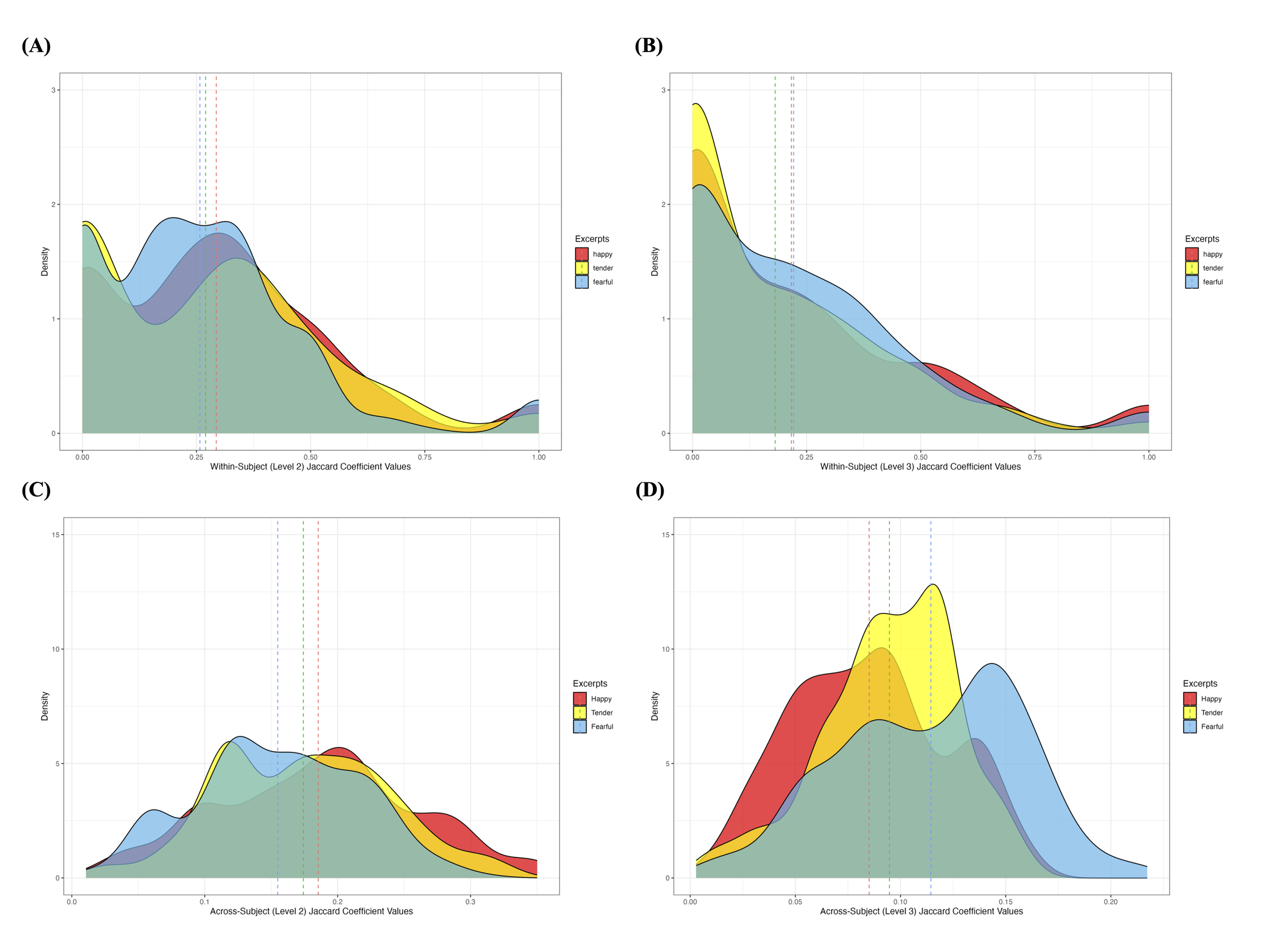


**Fig C. Comparing density distributions of consistency values of within- (across listening situations) and across-participant (across listeners) groups for each music excerpt type (with mean intercepts)**

(A) Level 2 within-participants consistency values. (B) Level 3 within-participants consistency values. (C) Level 2 across-participants consistency values. (D) Level 3 across-participants consistency values.

**
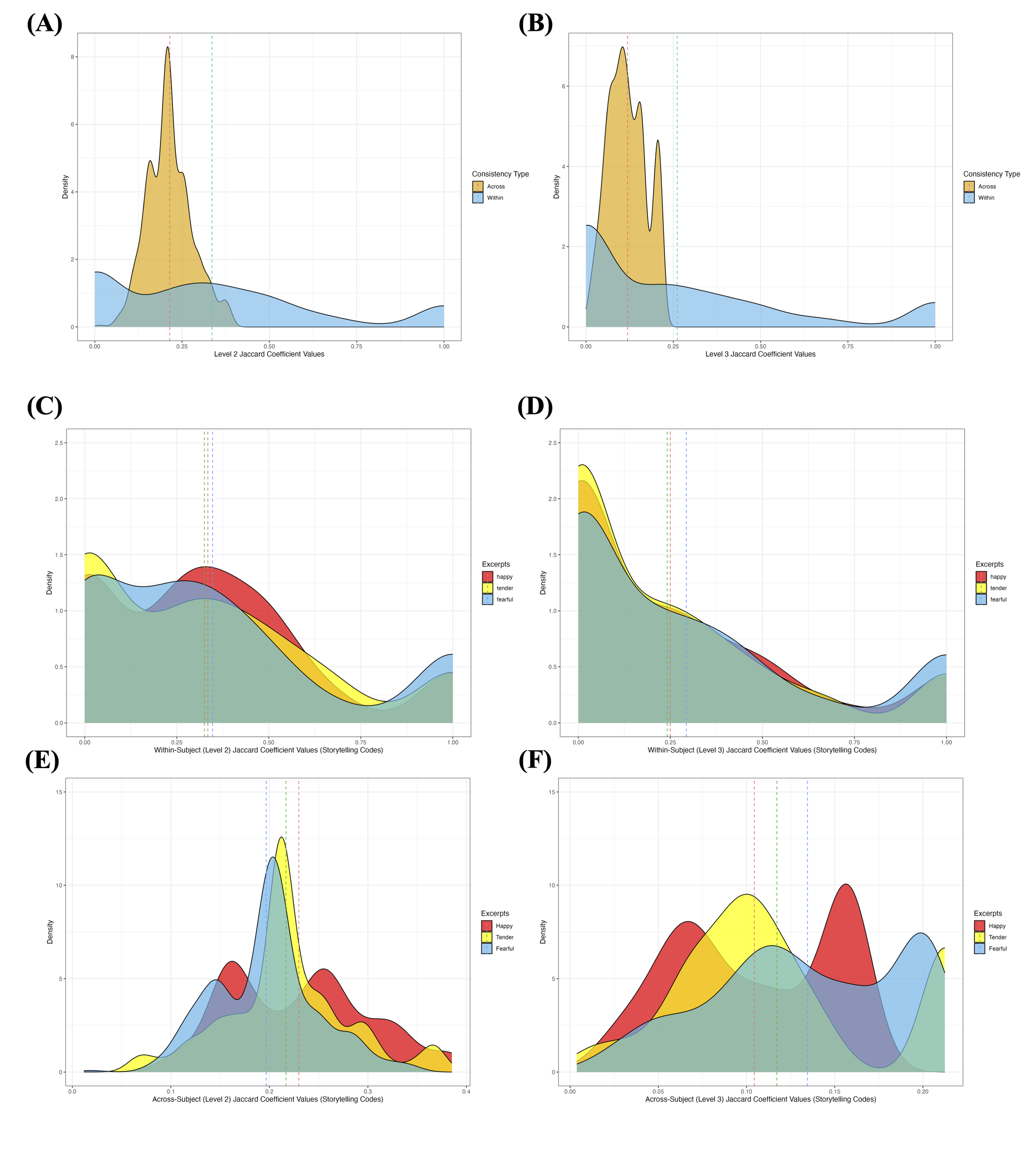
**

**Fig D. Comparing density distributions of consistency values of within- (across listening situations) and across-participant (across listeners) groups, as well as for each music excerpt type (with mean intercepts) for *Storytelling* codes**

(A) Level 2 *Storytelling* codes. (B) Level 3 *Storytelling* codes. (C) Level 2 within-participants consistency values. (D) Level 3 within-participants consistency values. (E) Level 2 across-participants consistency values. (F) Level 3 across-participants consistency values.

1. **Supplementary tables**

**Table A. Within-participant Jaccard consistency values including only Storytelling codes across levels 2 and 3 and across excerpts**

|  | Within-Participants | | | | | | | | |
| --- | --- | --- | --- | --- | --- | --- | --- | --- | --- |
|  | Level 3 (% of overall) | | | | Level 2 (% of overall) | | | | |
|  | **Overall** | Happy | Tender | Fearful | **Overall** | Happy | Tender | Fearful |  |
| 0% | **319 (44.3%)** | 110 (45.8%) | 109 (45.4%) | 100 (41.7%) | **217 (30.1%)** | 67 (27.9%) | 82 (34.2%) | 68 (28.3%) |  |
| 0.01–19.99% | **61 (8.5%)** | 18 (7.5%) | 25 (10.4%) | 18 (7.5%) | **42 (5.8%)** | 11 (4.6%) | 12 (5.0%) | 19 (7.9%) |  |
| 20–39.99% | **145 (20.1%)** | 50 (20.8%) | 48 (20.0%) | 47 (19.6%) | **194 (26.9%)** | 71 (29.6%) | 56 (23.3%) | 67 (27.9%) |  |
| 40–59.99% | **83 (11.5%)** | 29 (12.1%) | 24 (10.0%) | 30 (12.5%) | **133 (18.5%)** | 53 (22.1%) | 41 (17.1%) | 39 (16.2%) |  |
| 60–79.99% | **32 (4.4%)** | 10 (4.2%) | 12 (5.0%) | 10 (4.2%) | **47 (6.5%)** | 14 (5.6%) | 23 (9.6%) | 10 (4.2%) |  |
| 80–99.99% | **1 (0.1%)** | 1 (0.4%) | 0 (0.0%) | 0 (0.0%) | **0 (0.0%)** | 0 (0.0%) | 0 (0.0%) | 0 (0.0%) |  |
| 100% | **79 (11.0%)** | 22 (9.2%) | 22 (9.2%) | 35 (14.6%) | **87 (12.1%)** | 24 (10.0%) | 26 (10.8%) | 37 (15.4%) |  |

Within-participant total % calculated from N = 720. From total % for musical excerpts were computed using each individual N sizes; N_Happy_ = 240, N_Tender_ = 240, N_Fearful_ = 240.

**Table B. Across-participant Jaccard consistency values including only Storytelling codes across levels 2 and 3 and across excerpts**

|  | Across-Participants | | | | | | | | |
| --- | --- | --- | --- | --- | --- | --- | --- | --- | --- |
|  | Level 3 (% of overall) | | | | Level 2 (% of overall) | | | | |
|  | **Overall** | Happy | Tender | Fearful | **Overall** | Happy | Tender | Fearful |  |
| 0% | **0 (0.00%)** | 0 (0.00%) | 0 (0.00%) | 0 (0.00%) | **0 (0.00%)** | 0 (0.00%) | 0 (0.00%) | 0 (0.00%) |  |
| 0.01–4.99% | **99 (9.3%)** | 42 (11.9%) | 29 (8.2%) | 28 (7.9%) | **2 (0.2%)** | 1 (0.3%) | 0 (0.0%) | 1 (0.3%) |  |
| 5–9.99% | **322 (30.4%)** | 128 (36.3%) | 128 (36.3%) | 66 (18.7%) | **21 (2.0%)** | 1 (0.3%) | 14 (4.0%) | 6 (1.7%) |  |
| 10–14.99% | **308 (29.1%)** | 82 (23.2%) | 116 (32.9%) | 110 (31.2%) | **142 (13.4%)** | 23 (6.5%) | 39 (11.0%) | 80 (22.7%) |  |
| 15–19.99% | **168 (15.9%)** | 101 (28.6%) | 5 (1.4%) | 62 (17.6%) | **215 (20.3%)** | 113 (32.0%) | 46 (13.0%) | 56 (15.9%) |  |
| 20–24.99% | **162 (15.3%)** | - | 75 (21.2%) | 87 (24.6%) | **391 (36.9%)** | 64 (18.1%) | 169 (47.9%) | 158 (44.8%) |  |
| 25-29.99% | **-** | - | - | - | **182 (17.2%)** | 88 (24.9%) | 53 (15.0%) | 41 (11.6%) |  |
| 30-34.99% | **-** | - | - | - | **73 (6.9%)** | 45 (12.7%) | 17 (4.8%) | 11 (3.1%) |  |
| 35-40% | **-** | - | - | - | **33 (3.1%)** | 18 (5.1%) | 15 (4.2%) | - |  |

Across-participant total % were calculated from N = 1059. From total % for musical excerpts were computed using each individual N sizes; N_Happy_ = 353, N_Tender_ = 353, N_Fearful_ = 353.
