# Supplementary material for: Music listening evokes story-like visual imagery with both idiosyncratic and shared content
Source: PLoS One. 2023 Oct 26;18(10):e0293412. doi: 10.1371/journal.pone.0293412 (PMC10602345; doi:10.1371/journal.pone.0293412)
Supplement: Attachment — Submitted filename: PONE-D-23-03570 - REVIEW.pdf [file pone.0293412.s003.pdf]

Dear Dr. Markostamou,
Dear Authors,

I had the great pleasure to review the article '*Music listening leads to predominantly story-like visual imagery that shows idiosyncratic tendencies'* submitted for publication to PLOS ONE (PONE-D-23-03570; review request received: 18.02.2023, review accepted: 20.02.2023, review submitted: 22.02.2023).

This large-scale online study investigates inter- and intra-participant consistency of music-indued visual mental imagery. The manuscript is well written, the experiment is well designed to address the core research questions, and the results fill an important gap in the literature. The manuscript is a great contribution to the field and well suited to the audience of PLOS-One.

I can strongly recommend the manuscript for publication; however, I have a few minor suggestions and three larger concerns regarding formalism and statistical analysis that I recommend the authors should address first. I think the authors can easily address these in a revision.

**Major Comment**

1) The manuscript in general is of very high quality, however, the results section and statistical approach in its current state is not on par with the rest of the excellent manuscript. Despite the experimental design relying on various related variables that are co-dependent, the authors opted for a very simple analytical approach and base much inference on a large number of individual correlations and t-tests, which (if sticking to the Null-Hypothesis-Significance-Testing analytical frame worked use here) not only leads to a dramatic alpha error accumulation, but also misrepresents the data structure, whilst simultaneously being unnecessarily verbose. This is a shame, as the data set and results are very exciting and constitute a great contribution to the field. A slightly more appropriate analysis that focuses on the main narrative of (in)consistency in music-induced visual mental imagery would substantially increase the quality of the paper, make greater use of the valuable data the authors collected, and streamline the results section. I've provided some detailed suggestions, including annotated example code in the detailed comments.

2) The formal calculations of the 'between' consistency seem unconventional and possibly a bit off. The authors calculate three metrics of consistency. Within consistency, which is operationalised through Jaccard coefficient index between topic codes of the first and second free format responses, which makes a lot of sense. Across consistency, which is the averaged Jaccard coefficient between a given participant and each other participant in the same condition, which also makes a lot of sense. And a between consistency (largely used for the within vs between consistency comparison), which is the same as the across consistency, only that rather than exhaustively calculates, it samples a single other participant. I see no tangible advantage to the between measure over the across measure, but it does come with a lot of disadvantages (e.g., sample biases, lack of reproducibility, low statistical resolution, and reliance on a particular random seed that would need to be shared). It is not clear why this metric is needed or how it provides 'greater comparison' for within vs between tests. I recommend removing the between metric and instead focusing on the across consistency metric, which captures the same information, just much more reliably.

3) Comparing consistency levels across levels (i.e., Level 2 vs Leve 3) is formally problematic. This is because the results reported are an innate feature of the data modelling framework. Through the authors' thematic analysis, the data is structured as a convolutional, embedded hierarchy where each Level 3 codes projects to a point on the Level 2 space. A structure like this necessitates that consistency (as calculated but Jaccard coefficient indices) increases as you move from Level 3 to Level 2, and the factor of increase is largely determined by the distribution of converging coding labels, rather than semantically interpretable properties of participants' responses. I provide more detail on this in the detailed comments. I strongly recommend removing these analyses from the manuscript, both in the results section as well as their interpretation in the discussion, as this finding is simply an innate feature of your annotation strategy, and instead focusing on the comparison within levels (which are very exciting).

At the end of the detailed comment section, I propose an alternative statistical approach and structure to the results section for the authors to consider.

## Detailed comments

I know no one likes receiving a lot of comments, but I hope you take these as the constructive appreciation of your work, as they are intended:

**p.1: Title** *'Music listening leads to predominantly story-like visual imagery that shows idiosyncratic tendencies'*

**Comment:** I feel the title might be a bit too strong. You report (even on the detailed Level 3) 20.6% within and 9.8% across consistency. Considering that participants had the opportunity to imagine whatever they wanted, it is remarkable how close your 'across' consistency measure got to your 'within' consistency measure. A title along the lines of the following might more closely represent your numerical findings: *'Music listening evokes story-like visual imagery with both idiosyncratic as well as shared content'*. Of course, the title is your choice, so please consider this merely a suggestion.

**p.2, l. 31:** Abstract: *'Of the initial sample, 254 respondents completed the survey again three weeks later.*

**Comment:** The abstract is very well written and gives an engaging summary of the project. However, this particular sentence seems a bit out of place. At this point of the abstract, the reader does not know yet what the survey is. I recommend moving the sentence to a slightly later place. For example:

> *'We recruited an online sample (N = 353) who listened to three film orchestral music excerpts representing happy, tender, and fearful emotions. For each excerpt, listeners rated how much visual imagery they were experiencing and how vivid it was, their liking of and felt emotional intensity in response to the excerpt, and, finally, described the content of any visual imagery they may have been experiencing. Further, they completed items assessing a number of individual differences including musical training and general imagery ability. Three weeks later, participants were invited to complete the survey again. '*

**p.3, l.63: Introduction** *'However, while it is unlikely that music-induced visual imagery solely comprises of static mental pictures, little is known about how it may develop over time. '*

**Comment:** Could just be me, but I found the phrasing here a bit unclear. What does 'develop over time' refer to? Based on the content of the paper, I guess it is about test-retest reliability, but since the first part of the sentence is about static images, one might think 'develop over time' refers simply to dynamic images within one imagery session. The next sentence then jumps to across listener consistency. Maybe you can rephrase this sentence to emphasize your point.

**p.3, l. 64:** *'Indeed, to date, there has been no systematic investigation of visual imagery content during music listening and it is unknown the extent to which it is consistent across listeners. '*

**Comment:** I think this might be a bit too strong. There are a few papers that look at across participant consistency of mental imagery in general, and some in music, and I think discussing this might be useful here. For example:

*Mitchell, D. J., & Cusack, R. (2016). Semantic and emotional content of imagined representations in human occipitotemporal cortex. Scientific reports, 6(1), 1-14.*

Reddy, L., Tsuchiya, N., & Serre, T. (2010). Reading the mind's eye: decoding category information during mental imagery. *Neuroimage*, *50*(2), 818-825. (both papers look at it from a neuroscientific perspective; neural encoding similarities can be used for object classification)

*Dijkstra, N., Hinne, M., Bosch, S. E., & Van Gerven, M. A. J. (2019). Between-subject variability in the influence of mental imagery on conscious perception. Scientific reports, 9(1), 1-10.* (who look at it from a priming perspective; similarities in efficacy of imagined primes suggests similarities in imagined objects)

*Bigelow, E. J., McCoy, J., & Ullman, T. (2022). Non-Commitment in Mental Imagery.* (Similarity between non-commited features of the imagined content, suggests similarities in imagined content)

And there are a few who (at least partially) consider this in the specific case of Music listening:

*Margulis, E. H., Wong, P. C., Turnbull, C., Kubit, B. M., & McAuley, J. D. (2022). Narratives imagined in response to instrumental music reveal culture-bounded intersubjectivity. Proceedings of the National Academy of Sciences, 119(4), e2110406119.* (which you also discuss later on)

*Kaelen, M., Roseman, L., Kahan, J., Santos-Ribeiro, A., Orban, C., Lorenz, R., ... & Carhart-Harris, R. (2016). LSD modulates music-induced imagery via changes in parahippocampal connectivity. European Neuropsychopharmacology, 26(7), 1099-1109.* (psychedelic drugs affect music-induced imagery similarity across tested participants, highlighting similarities in music-induced imagery implementation)

*Dahl, S., Stella, A., & Bjørner, T. (2022). Tell me what you see: An exploratory investigation of visual mental imagery evoked by music. Musicae Scientiae, 10298649221124862.*

The Dahl et al (2022) paper in particular discusses similarities and differences in music-induced mental imagery content in great detail.

It might be worth discussing some of the references above and to be a bit more specific about the exact gap in the literature you are referring to here, currently the statement reads a bit too broad.

**p. 3. L.68-71**: *"It is also unclear how certain relevant individual differences (such as musical training, general visual imagery ability, synesthetic tendencies or participation in the visual arts) may be associated with prevalence, vividness and consistency of music-induced visual imagery. "*

**Comment:** The main motivation statement here reads like 'we don't know this yet, so it's worth investigating'. Personally, I absolutely agree with the sentiment, however, PLOS ONE is a generalist's journal, so I think it would be important to strengthen the motivation here a bit. From a big picture perspective, what can we learn from exploring this topic in greater detail, what are the implications for our understanding of human cognition, or the role of culture and society that different results would suggest. I think your study is very exciting with many large implications and I think here is the place to highlight a bit further why investigating this topic is important, and why it is worth for the readers of PLOS ONE to read the rest of your exciting paper. Additionally, the unique selling point and main narrative of the paper is about within vs across consistency, so to streamline the narrative, it might be better focus on*"It is also unclear how certain relevant individual differences (such as musical training, general visual imagery ability, synesthetic tendencies or participation in the visual arts), imagery vividness and prevalence may be associated with consistency of music-induced visual imagery*. (That is focusing on consistency as the main dependent variable of interest, but this is just a suggestion to make your narrative flow nicer)

**p. 4, l. 75:** *'In a similar vein, while visual imagery has been associated with aesthetic and emotional engagement with music, the evidence of such links remains limited. '*

**Comment:** Maybe cite one or two of the theoretical frameworks or recent review articles that conceptualize this in detail here. For example:

Juslin, P. N. (2013). From everyday emotions to aesthetic emotions: Towards a unified theory of musical emotions. *Physics of life reviews*, *10*(3), 235-266.

Taruffi, L., & Küssner, M. B. (2022). Visual Mental Imagery, Music, and Emotion. In *Music and Mental Imagery*.

**p. 8, l. 176** *"…within, across, and between..'*

**Comment:** The conceptual difference between 'across' and 'between' is not really clear here. As mentioned earlier and detailed later, the formal difference is also not clear, so I suggest dropping the 'between' measure entirely.

**p. 9, l. 197** *"Further, we predicted we would find little difference between synaesthetes and non-synaesthetes in the consistency of their visual imagery experience; this is due to previous results stating that non-synaesthetes are capable of showing consistency in a colour-picking for letters task, performance on which was predicted by scores in a visual imagery task [39]. '*

**Comment:** I find the logical flow here not very clear. You predict no differences between two groups, but use a statistical approach that is not capable of supporting the Null-hypothesis, only rejecting it. If you are interested in supporting the Null-hypothesis, you'll have to use Bayesian statistics, but frequentist/NHST approaches like the ones you use in your analysis lack the ability to support the null hypothesis (i.e., not rejecting the null is not equal supporting the null). Additionally, synaesthetes is a very broad term, maybe be clear about which precise conditions you are interested in (e.g., all those that have co-evoked percepts involving either sound or visuals or both?), otherwise it isn't clear why you would consider individuals that say, have co-evoked percepts of pain and smell. Furthermore, why would you predict a lack of an effect between synaesthetes vs non-synaesthetes solely based on the finding that non-synaesthetes can do a color-picking task consistency that has some degree of correlation with visual imagery task. I feel like I'm missing something, but currently the logical flow here feels a bit shacky.

**p.10 l. 223. Materials and Stimuli**

**Comment:** Most readers will be unfamiliar with the film-music corpus from Eerola & Vuoskoski and will likely assume that these songs are reasonably familiar to at least some participants. The corpus contains published film music, judged to be relatively unfamiliar by a few raters, but some of the pieces will likely sound familiar to participants (e.g., the Vertigo soundtrack is part of that corpus). I recommend providing a bit more information about the pieces you selected, maybe indicate which exact soundtracks the three selected pieces are from.

**p.12, l.265:** *'They were advised to pay attention to any visual imagery that they may be experiencing..''*

**Comment:** In your instructions you very much highlight visual imagery, which makes a lot of sense given your research questions. However, there is a lot of literature pointing towards the effects of eyes open vs close in visual mental imagery, both in general:

*Vredeveldt, A., Hitch, G. J., & Baddeley, A. D. (2011). Eyeclosure helps memory by reducing cognitive load and enhancing visualisation. Memory & Cognition, 39(7), 1253– 1263. https://doi.org/10.3758/s13421-011-0098-8*

*van den Hout, M. A., Engelhard, I. M., Beetsma, D., Slofstra, C., Hornsveld, H., Houtveen, J., & Leer, A. (2011). EMDR and mindfulness. Eye movements and attentional breathing tax working memory and reduce vividness and emotionality of aversive ideation. Journal of Behavior Therapy and Experimental Psychiatry, 42(4), 423–431. https://doi.org/10. 1016/j.jbtep.2011.03.004).*

And in music in particular:

*Hashim, S., Stewart, L., & Küssner, M. B. (2020). Saccadic eyemovements suppress visual mental imagery and partly reduce emotional response during music listening. Music & Science, 3, https://doi.org/10.1177/2059204320959580*

*Herff, S. A., McConnell, S., Ji, J. L., & Prince, J. B. (2022). Eye Closure Interacts with Music to Influence Vividness and Content of Directed Imagery. Music & Science, 5,* https://doi.org/10.1177/2059204322114271

Were you participants instructed to keep their eyes open, or to close them? Or was it up to them? I think this is worth mentioning, and maybe discussing in a couple of sentences in the discussion as the simultaneous visual input (or absence of it) can have dramatic effects (the effect are so large, they are used in therapeutic contexts) and this has been explored in particular in the context of – and interaction with- music-induced imagination – the present focus on visual mental imagery warrants a short discussion about this.

**p.3.3 Annotations**

**Comment:** The data annotation approach makes a lot of sense and was clearly diligently done. Coding all the responses must have been a difficult task, and I just wanted to thank the authors and annotators for their great contribution. Making this dataset publicly available will be very valuable to the field.

**p. 15: Results** *'Across-participants was computed by comparing the list of themes from each individual participant with the lists from every other individual in the sample, resulting in values per participant that were then averaged to create a single consistency value per individual. '*

**Comment:** The general approach is solid, but if you have 352 values per participant (prior to averaging), then this means that you must have averaged across the three pieces. Or did you calculate the Jaccard coefficient index separately for each of the pieces? I think calculating the coefficient separately for each of the three pieces and each participant would make the most sense given your data structure, this would allow you to visualise and test the within vs across-distributions thoroughly and provide very insightful results, however, it isn't clear from the description which approach was taken.

**p. 15. l, 344:** *'Finally, to allow greater comparison of consistency values within and between individuals, a between-participants consistency was calculated by comparing each participant with a random other individual. With regard to across- and between-participant consistency specifically, individuals were always compared against other individuals' responses that were within the same excerpt type.*

**Comment:** This part really confused me. Based on your description, you are effectively producing a low-resolution, sampled metric from your across-participant measure. I do not understand in what way this allows greater comparison. If anything, it allows less reliable comparison, because your comparison would be massively influenced by the particular dyads you are sampling. In addition, this approach is effectively not reproducible unless you share the particular random seed used to pair the dyads, and there is no way of checking whether the dyads were handpicked. Of course, I know that they weren't, but this approach would theoretically allow it, so this is more a point about general rigor and reportability. I really do not see any advantage in this metric over your across metric. Currently, it feels like you are throwing away a great amount of your precious data without gaining anything in return. Long story short, I strongly suggest ditching the 'between' metric and focusing on within vs across. This will provide very compelling support for your project. At the end of the detailed comments section, I'll propose an alternative structure and analytical approach for your results, you can take as much or little of it on board as you like, but I think it is worth considering.

**p. 17-19** *'Out of all the ratings of the 353 responds collapsed across the three excepts'*

**Comment:** Why do you collapse across the three pieces? You are throwing away a lot of data. Additionally, the large number of Pearson correlations you are conducting afterwards result in a dramatic alpha error accumulation. If you were to correct for this, basically no result would be significant anymore (I do not suggest you do this). Additionally, based on the degrees of freedom, it seems like all your correlations were also calculated on the aggregated (across excerpts) data even though you deliberately chose those songs to be different across key dimension (such as emotional intensity, or might differ in liking), so you are again throwing away a large amount of data (in the order of magnitude factor 3!). In many instances it isn't clear why you are correlating particular variables (say liking and emotional intensity) when there wasn't a clear hypothesis for this. Additionally, the multiple correlations with your target variables 'e.g., imagery vividness' using variables that show co-linearity, such as emotional intensity and liking, makes interpreting these results effectively impossible, as all your effects may or may not be tapping into the same variance.

Long story short, I recommend deleting all in-text correlation reporting in the 4.1 section, keep the correlation table for interested readers, and then run a quick model (one for each of your literature guided hypothesis, and ignore those for which you have no literature motivation) for your actual inference that accounts for all these dependencies. This will shorten this section, make the results more interpretable and readable, will make full use of your statistical power, and will be cleaner in terms of avoiding violations of independence. At the end of the detailed comments section, I'll provide additional detail that might be useful.

**p. 26-29.** *'How consistent is visual imagery and to what extent does it depend on abstraction'*

**Comment:** As mentioned above, is see a formal issue in analysing and making claims about increases in consistency between Level 2 and Level 3, and suggest deleting these from the results and discussion.

This is because the topography of convulsionary models dictates the 'consistency' is inherited, regardless of whether you build these structures on themes, topics, classes, or categories.
Your very useful online supplemental material helps makings this clear: The level1.level2.level3 coding structure highlight that this is indeed one of the type of structures that has this issue.

This point might be intuitive to you, but just to make sure that we are not talking cross-purposes, here is a reduced simplified example with a convulsionary hierarchical classifier that has the same issue: Imagine I like (object codes in brackets):
**Apples** (1(food).1(fruit).1(apples),
**Oranges**(1.1.2),
**Pizza**(1.2(Baked goods).1, and
**Bananas**(1.1.3),

and you like
**Passionfruit**(1.1.4),
**Strawberries**(1.1.5),
**Cake**(1.2.2), and
**Oranges**(1.1.2). Now we calculate the (simplified) Jaccard Coefficient (defined as shared divided by all distinct codes) on level 3:
1 shared over 7 distinct codes combination = ~.14

Then, we move up one level to level 2, which effectively projects this through a convolutionary layer, Everything that was shared before, is still shared, but we additional share a few more food favourites (Fruits, baked good, we now have 100% shared food liking). As you go up the levels, the sharedness (or consistency) can only increase whilst the total number of labels can only decrease, which means the Jaccard Coefficient keeps going up. As a result, cross level comparison doesn't make much sense, as much of the results you would draw from this is dependent first and foremost on the exact convolution structure you have.
(There are ways of partialling out this convergence, but they get very complicated, very quickly, so I'd advise against this).
However, looking how high consistency within vs across is on Level 2 and Level 3 separately makes a lot of sense and is very useful, because it shows that even on the detailed level 3, a sizeable portion (~10%) of coding

labels is shared, so it's great and very useful you report this. As mentioned before, at the end of this detailed comment section, I provide some additional suggestions on restructuring your results section.

**p. 29 Table 3**

**Comment:** This is a very useful table, thanks a lot for this.

**p. 33. Discussion**

As mentioned before, I strongly advise removing all level 3 versus level 2 consistency comparisons, instead, you could just focus on discussing the overall findings that hold even in level 3, which is an interesting pattern of shared and idiosyncratic content. Additionally, if you focus on across vs within and remove the between measurement, then the discussion would also require some adjustment but become much more streamlined in the process.

**p. 35, l. 703** *'At first glance, our finding show notable differences with a recent investigation by Magulis et al. [6]*

**Comment:** Agreed, the overall pattern is the same (higher consistency within person/culture, lower consistency across person/culture, with some degree of overlap remaining). The main difference is not in the results, but the metric, one focusing on continuous similarity on a latent variable (cosine similarity) one focus on more discreet topic overlap (yours). Both have their advantages and disadvantages. Cosine similarity allows continuous weighting of similarity, but doesn't allow exploring distinct topic prevalence or absence. Your approach allows identifying specific themes, but you lose the ability to weight topic importance. Long story short, Margulis measures similarity, you measure whether (amongst others) two reports cover the same topic, regardless of how intense. As a result, I would recommend being a bit more caution with your statement on **p. 36, l..714** *'It was also a more valuable method with which to approach our dataset'.*

Your method is great and very useful for your research question, but not necessary inherently 'more valuable' for the dataset perse.

**p. 38, l. 764** *'First, our analyses show that emotional intensity is related to across-participants consistency hinting at the idea that part of what determines consistency across individuals may be a shared high level of emotion.'*

**Comment:** Very interesting finding! In general, the 5.3 section is very well done.

**p. 39, l. 802** *"Musical training did however show a very weak but negative significant relationship with music liking, which could suggest that those with training in music may be mildly uninterested in film music. "*

**Comment:** This is a bold conclusion in general, but in particular considering that you've only tested three songs and averaged across them. I would recommend just deleting this.

**p. 3, l. 805:** *'Our results confirmed our expectation that those who experience a form of synaesthesia are no different in the ways they experience prevalence and vividness of visual imagery across music excerpts and in response to individual listening tracks.'*

**Comment:** I would recommend being more careful with your interpretations on synaesthesia. This is a condition with dramatic differences between individuals, you've relied on self-report, without additional information about which senses co-create percepts, and your statistical approach (NHST) by definition cannot support the null-hypothesis, only reject it (only a Bayesian Framework can support the Null). So maybe simply rephrase this along the lines of:

> *''As with previous literature, we have not observed evidence supporting differences in self-reported synaestheses to differ in their prevalence or vividness of visual imagery.''*

**p. 40, l '820':** *"Thus, although the directionality is unclear, increased participation within various art modalities (in this case, visual) may increase imagery engagement with music. "*

**Comment:** Also a very exciting finding! However, maybe rephrase is slightly, as it is not only a question of directionality (A -> B vs B->A), but it could also be a question an entirely different sets of variables being responsible that affect both (e.g., C -> A & C ->B). Maybe a phrasing like *"Thus, although the general chain of causality is unclear, increased participation within various art modalities (in this case, visual) may increase imagery engagement with music. "*

**p. 40, l. 831:** *'The increase in correlation between across-participant consistency and the majority of behavioural ratings and individual differences could point to shared cultural and societal influences, despite our sample being sourced from a wide range of backgrounds. "*

**Comment:** I'm not sure I get this, but it might just be me. Which correlations are increasing? Wouldn't that require an interaction term? And why does the point towards a shared cultural and societal influence? Maybe you can rephrase and elaborate a bit here.

**p. 40 l.83:** *"The similar levels between within-participants consistency and ratings that we found may indicate…''*

**Comment:** Same thing, I struggle parsing the sentence. Is this about narrow distributions of all rating scales and the within-participant consistency? I'm not sure, maybe rephrase and elaborate.

**Online supplement:**

These are very useful, thanks a lot! I think the survey1_ratings and survey2_ratings files still contain participants' prolific IDs in the anon-id column, in addition to the anonymized 'ID' column, you might want to remove those.

**Suggested Statistical Approach and Results Structure**

As mentioned before, in the following I'll suggest a different structure to the results section and statistical approach that I believe addresses all the concerns raised above and would further improve the quality of your work. This is just one of the many ways on how to address the aforementioned concerns and it is entirely up to you to choose your pathway, but I thought it might be useful for your to see one possibility spelled out in detail rather than me just nagging about things without offering solutions.

**Part 1: Start with the Thematic analysis of music-induced visual imagery (current section 4.2)**

**Part 2: Make your strong point about within vs across (this is the unique selling point of your excellent work and should be the focus of all sections):**

(Starting with Level 2 codes):

1) Calculate **within** Jaccard coefficient index, separately for each participant and each song. This will result in 3 distinct within distributions, one for each song. Each distribution containing 254 data points.
2) Calculate **across** Jaccard coefficient index, separately for each participant and each song. This will result in another 3 distinct across distributions, one for each song. Each distribution containing 353 data points.
3) Plot the distributions, preferably in a continuous density plot rather than a discrete histogram which can be misleading based on bin-size.
4) Test within vs across in one of two ways, either:
    a. Simply test for each song the within vs the across distribution. You can do this with your KS-test, or using symmetric KL-divergence (faster, because it's close to what you already do, however not quite as elegant as option b and would require 3 tests, one for each song). OR
    b. even more elegantly, used a linear mixed effect model. In your method section, you mentioned you use R as your environment of choice, so in R, simply create a data frame (DF) in long-format containing all your data from both surveys (up to you if you only want to include participants that were available at both time points, but theoretical the model is robust to deal with the missing data of the participants that were only there once). That is, six rows per participant (3 rows containing the 3 within coefficient for the different songs, and 3 rows containing the within coefficient for the different songs. If you include the participants that only did one survey, then those will not have the 3 within rows, which is fine, the model is robust to cope with this because we have a random participant intercept). Your columns are 'ID',''Coefficient", 'AcrossVsWithin', 'Excerpt' and you run in R a simple mixed effects models using the lme4 package (here is a straightforward tutorial https://jontalle.web.engr.illinois.edu/MISC/lme4/bw_LME_tutorial.pdf)

    #model formular in R:

    library(lme4) #you might need to install the package first as well as its dependencies.

    modelFit <- lmer(Coefficient ~ AcrossVsWithin+(1|ID)+(1|Excerpt), data=DF)

    #This predicts the Jaccard coefficient based on whether the coefficient came from the within or across comparison, whilst controlling for cross random effects of participant and stimulus. Now you are making full use of all your data simultaneously which increases your statistical power by ~factor 6, and you are much more closely representing your data structure and are not violating any statistical assumptions whilst also avoiding alpha error accumulation, because you fit everything in light of each other.

    summary(modelFit)

    #the AcrossVsWithin factor will answer you research question in one elegant model that truthfully represents your data structure, whilst accounting for both participant and excerpt random effects, and makes full use of the statistical power of your data set (~1524 observations, rather than 254, whilst accounting for data-dependency structure). You can then report your entire results in one short phrase, whilst still making much greater use of the fantastic data you've collected, and you don't need to split by song anymore.

5) Repeat step 1-4 with level 3 codes, impressing everyone that the results largely stay the same in the higher granularity.

**Part 3: After convincing everyone of your interesting pattern on within vs across consistency, follow up with explorative analyses to explore sources of where those differences may come from**

1) Based on your literature review, and your main narrative, your data is structured in 2 key dependent variables (within consistency, across consistency), and a large number of variables related to

participant information that may influence your dependent variables (Music liking, emotional intensity, VVIQ, Musical Training, VisualArts, vividness, and prevalence). Rather than testing all combinations as you are currently doing, I suggest you include the correlation table as a form of general description of your data structure (so the reader can pick out what they are interested in). But for the actual inference for the present work, you focus on the combination that are essential for your main narrative which is *'How consistent is music-induced imagination between and across participants, and which factors contribute to it''*

2) That means, you can simply structure your data in R in long format (DF2), with

   Columns (ID, WithinConsistency, AcrossConsistency, Liking, EmotionalIntensity, VVIQ, Musical Training, vividness, Excerpt).

   This means you have 3 rows for each participant, with some participants having NAs in the within column (because they only participated in one session)

3) Simply run two models:

```
library(lme4) #you might need to install the package first as well as its dependencies

modelFitWithin <- lmer(WithinConsistency ~ Liking+
EmotionalIntensity+VVIQ+MusicalTraining+Vividness+(1|ID)+(1|Excerpt), data=DF)

#If you want to add additional predictors, you can simply throw them in here as well like
+SynaesthetiaYesNo+VisualArtsBackground etc and fit every effect in light of the others

summary(modelFitWithin)

modelFitAcross <- lmer(AcrossConsistency ~ Liking+
EmotionalIntensity+VVIQ+MusicalTraining+Vividness+(1|ID)+(1|Excerpt), data=DF)

summary(modelFitAcross)
```

   The coefficients of your fixed effects (Liking+ EmotionalIntensity+ VVIQ+ MusicalTraining+Vividness etc) then tell you whether they have a significant effect on your dependent variables (within or across) whilst controlling for cross-random effects of participants and excerpt, and whilst considering the partial effects of the respective other variables. You can also obtain p-values for each coefficient (see 'pbkrtest' package). This makes full use of all the data you've collected, avoids violations of independence, avoids alpha error accumulation, and nice and clean tells you what you need to know. I think your results section would improve a lot if you focus on your main narrative of consistency, rather than trying to test and interpret independently every single correlation in your data. The same goes for the Discussion section, which then would have a nice narrative following your results a) Interesting themes identified b) Interesting idiosyncratic and shared consistencies observed c) some interpretable sources for these (in)consistencies.

Thank you very much for your great contribution. I thoroughly enjoyed the manuscript and think it offers great insights and the dataset itself is fantastic additional contribution. I understand that the comments on the results section might feel a bit frustrating, but I do think they are important and will help in further improving your manuscript so that it will get the recognition and attention that it deserves. I'm looking forward to reading a revised version and seeing this researched published.

Signed,

Steffen Herff
