# Supplementary material for: Music listening evokes story-like visual imagery with both idiosyncratic and shared content
Source: PLoS One. 2023 Oct 26;18(10):e0293412. doi: 10.1371/journal.pone.0293412 (PMC10602345; doi:10.1371/journal.pone.0293412)
Supplement: Attachment — Submitted filename: PONS review.docx [file pone.0293412.s004.docx]

PONS review

The paper: ”Music-listening leads to predominantly story-like imagery…” is a very accurate and analytical investigation of visual imagery provoked by listening to music, that cannot be qualified as systematic due to the limitation of the genre of the musical stimuli used and of the emotions considered.

The general aim is four-fold: To confirm literature about the association ”between visual imagery, emotion, and aesthetic appeal”; to find the prevalent themes induced by music in visual imagery; to assess their consistency within, across, and between individuals; to find potential factors the influence this consistency.

In this highly analytical number paper, I will list some minor required precisions in the order of appearance.

Lines 210-218: It would be useful to present the different types of participants in a table, also distinguishing Males and Females. This distinction should also be investigated in the results, all the paper along.

Lines 228-9: the number of the reference should stay after the test name (i.e. after Inventory).

Line 267: a definition of the term “prevalence” is needed.

Lines 272-3: the order “associated with visual arts,” and synesthesia is the inverse of that indicated in the previous paragraph at lines 250.1.

Lines 311-3: Please, give more details about the way this hierarchical categorization was performed.

Line 410: Insert (VA) dopo visual arts and before the coma.

Lines 556-7: I think that the indication as first result (as already before in the aims, and afterward line 676) that “visual imagery experience was very prevalent” goes without saying, since according to my comprehension, visual imagery was expressly indicated as the subject’s task. Or were the commitments differently expressed? More interesting is the following indication…

Lines 686-8:…that visual imagery was accompanied by cross-modal aspects, aesthetic evaluation, and emotions, positively related to images vividness.

Lines 856-860: I think that the novelty of the methodological approach should be stressed as an important result of the research besides the…:

Lines 863- 964:… very well indicated key limitations.

Lines 906-918: Given all that is said above the Conclusion should be rewritten in order to put in evidence the bring-home results.
