# Supplementary material for: Music listening evokes story-like visual imagery with both idiosyncratic and shared content
Source: PLoS One. 2023 Oct 26;18(10):e0293412. doi: 10.1371/journal.pone.0293412 (PMC10602345; doi:10.1371/journal.pone.0293412)
Supplement: Attachment — Submitted filename: Response to Reviewers.docx [file pone.0293412.s006.docx]

Dear Editor,

Thank you for the opportunity to submit a revised version of our manuscript ‘*Music listening evokes story-like visual imagery with both idiosyncratic and shared content*’ to PLOS ONE. We are grateful to the reviewers for their constructive comments and suggested amendments to our paper. All changes are made on a revised version of the manuscript with tracked changes implemented (entitled ‘Revised Manuscript with Tracked Changes’) as well as in an additional revised unmarked version (entitled ‘Manuscript’), as requested.

Please find below the reviewers’ comments and our responses following each one:

**Comments from Reviewer 1 – Dr Steffen A. Herff**

**Comment:** *One final minor suggestion is to include the topic distributions (currently in-text in 4.1.1.-4.13) also in brackets behind the respective topics in Table 2 during the author proof stage. This would provide readers with a very comprehensive understanding of the topic structure when consulting Table 2. But this suggestion is entirely personal preference, and up to the authors to decide.*

**Response:** We thank the reviewer for their suggestion. The L1 and L2 topic distributions, originally only presented in the main text, have now also been included in Table 2 next to their corresponding theme label.

**Comments from Reviewer 2 – Dr Julian Cespedes-Guevara**

*I only have two small suggestions that I think could slightly enhance the readability of the paper:*

**Comment:** *1) I suggest signalling the hypotheses when reporting each analysis in the results section. For instance, on pages 25, lines 521 onward, the authors could mention that "In order to test hypothesis 2, we asked whether there were differences in consistency when comparing and individual with themselves (...)" etc. Similarly, in the first two paragraph of page 31, they could mention that the models confirm hypotheses 3, 4, 5 and 6, etc.*

**Response:** We agree with the reviewer that signalling the individual hypotheses across the Results section would provide more clarity. This has been implemented throughout this section as suggested.

**Comment:** *2) On Page 30, Table 5, I suggest including the aggregated proportions in the table. These are only suggestions; I leave it to the authors to decide whether incorporating them or not. There’s no need for me to review the paper once more.*

**Response:** We agree with this change and have included the aggregated values within the table alongside the individual musical excerpt values (see Table 5). Accordingly, the paragraph preceding this table that originally described the aggregated proportions has been rephrased to describe the table as a whole instead, as follows: ‘Table 5 presents a summary of visual imagery prevalence and vividness ratings divided and aggregated by musical excerpt. These values demonstrate high proportions of visual imagery prevalence and vividness across all musical excerpts, that persist even when one considers the individual excerpt types. Higher prevalence and vividness levels of visual imagery can be seen in response to the Fearful excerpt than the Happy and Tender excerpts, which both exhibit almost equal proportions.’.

**Comments from Reviewer 3 – Prof Marta Olivetti Belardinelli**

**Comment:** *As regards the gender differences in music and language processing (both implied in the visual imagery task of this research) there is plenty of research assessing it. See for example some of the first studies:*

- *Koelsch S, Maess B, Grossmann T, Friederici AD. Electric brain responses reveal gender differences in music processing. Neuroreport. 2003 Apr 15;14(5):709-13. doi: 10.1097/00001756-200304150-00010. PMID: 12692468.*
- *Koelsch S, Grossmann T, Gunter TC, Hahne A, Schröger E, Friederici AD. Children processing music: electric brain responses reveal musical competence and gender differences. J Cogn Neurosci. 2003 Jul 1;15(5):683-93. doi: 10.1162/089892903322307401. PMID: 12965042.*
- *Koelsch S, Gunter TC, Wittfoth M, Sammler D. Interaction between syntax processing in language and in music: an ERP Study. J Cogn Neurosci. 2005 Oct;17(10):1565-77. doi: 10.1162/089892905774597290. PMID: 16269097.*
- *Koelsch S. Music-syntactic processing and auditory memory: similarities and differences between ERAN and MMN. Psychophysiology. 2009 Jan;46(1):179-90. doi: 10.1111/j.1469-8986.2008.00752.x. Epub 2008 Nov 21. PMID: 19055508.*
- *Carrus E, Pearce MT, Bhattacharya J. Melodic pitch expectation interacts with neural responses to syntactic but not semantic violations. Cortex. 2013 Sep;49(8):2186-200. doi: 10.1016/j.cortex.2012.08.024. Epub 2012 Sep 20. PMID: 23141867.*

*According to my meaning, it is therefore important to cite the topic as a limitation of the study tied to the manuscript length, or as a feature research goal, as you prefer.*

**Response:** We thank the reviewer for their suggestion and useful resources. We have now included a paragraph in the *Implications, limitations, and future directions* subsection briefly addressing this, including additional references to clarify our points, as follows:

‘The design of the current study required participants to provide unrestricted reports on the content of their visual imagery to music, implicating their ability to verbalise their visual imagery experiences (i.e., constructing their narrative to music [4]). It is generally well evidenced that music and language are reflected by overlapping electrophysiological correlates [16,63–67], but some studies identify differences in this regard (e.g., between males and females [68–71], and as a function of musicianship [72–77]). Neuroscientific studies on music-induced visual imagery are only beginning to emerge (see [61,78] for first evidence of neural signatures). However, we suggest that future studies may seek to combine approaches like those taken in our current paper with emerging insights into neural underpinnings in order to advance knowledge of both the brain and visual imagery during music listening.’

We look forward to hearing from you soon regarding our submission and we are happy to respond to any further questions and comments that you or the reviewers may have.

With best regards, and on behalf of the co-authors,

Sarah Hashim
